# miR-363-3p inhibits tumor growth by targeting PCNA in lung adenocarcinoma

**DOI:** 10.18632/oncotarget.15448

**Published:** 2017-02-17

**Authors:** Yahong Wang, Ting Chen, Haili Huang, Yun Jiang, Lawei Yang, Ziying Lin, Huijuan He, Tie Liu, Bin Wu, Jie Chen, David W. Kamp, Gang Liu

**Affiliations:** ^1^ Clinical Research Center, Affiliated Hospital of Guangdong Medical University, Zhanjiang, China; ^2^ Department of Respiratory Medicine, Affiliated Hospital of Guangdong Medical University, Zhanjiang, China; ^3^ Department of Cardiothoracic Surgery, Affiliated Hospital of Guangdong Medical University, Zhanjiang, China; ^4^ The First Affiliated Hospital, Medical School of Xi’an Jiaotong University, Xi’an, China; ^5^ Department of Medicine, Northwestern University Feinberg School of Medicine and Jesse Brown VA Medical Center, Chicago, IL, USA

**Keywords:** miR-363-3p, lung adenocarcinoma, tumor growth, m-TOR/ERK signaling pathway, proliferating cell nuclear antigen (PCNA)

## Abstract

Increasing evidence suggests that microRNAs play key roles in lung cancer. Our previous study demonstrated that microRNA 363-3p (miR-363-3p) is downregulated in lung cancer tissues. In this study, we demonstrated that overexpression of miR-363-3p inhibits the proliferation and colony formation of A549 and H441 cells, while silencing of miR-363-3p has the converse effects. The anti-oncogenic function of miR-363-3p was verified in a mouse tumor xenograft model. Furthermore, cell cycle analysis showed miR-363-3p can induce S phase arrest by downregulating Cyclin-D1 and upregulating Cyclin-dependent kinase-2 in lung adenocarcinoma cells. Additionally, miR-363-3p enhances cell apoptosis, whereas miR-363-3p inhibitor prevents apoptosis and leads to downregulation of Bax and Bak expression. The anti-proliferative function of miR-363-3p toward lung cancer cells may be explained by its ability to inhibit the activation of the mTOR and ERK signaling pathways. Using target prediction software and luciferase reporter assays, we identified PCNA as a specific target of miR-363-3p. miR-363-3p can decreased the accumulation of endogenous PCNA in lung adenocarcinoma cells. Moreover, exogenous expression of PCNA relieve the inhibition of miR-363-3p on cell proliferation, colony formation and mTOR and ERK signaling pathways. Taken together, our data indicate that miR-363-3p suppresses tumor growth by targeting PCNA in lung adenocarcinoma.

## INTRODUCTION

Non-small cell lung cancer (NSCLC) is the most common cancer worldwide and accounts for approximately 80% of the total lung cancer cases [1, 2] The poor prognosis of NSCLC is due to late disease presentation, tumor heterogeneities within histological subtypes, and the relatively limited understanding of tumor biology [3, 4]. Emerging targeted therapies directed against specific cellular alterations require precise sub-classification of NSCLC that is beyond the capabilities of standard histopathological diagnostic techniques [5, 6]. Therefore, the identification of new approaches for classifying and treating NSCLC is critical for understanding this deadly disease.

MicroRNAs are small molecules of about 22 nucleotides. Accumulating evidences demonstrates that miRNAs influence the development and progression of cancer by modulating the expression of key regulators in proliferation, metastasis and invasion [7–11]. miR-363 is a recently identified tumor suppressor in various cancers, including hepatocellular carcinoma, breast cancer, head and neck cancer, gastric cancer, colorectal cancer and neuroblastoma. miR-363 regulates tumorigenesis, metastasis, invasion and chemotherapy-induced apoptosis by targeting crucial factor, such as S1PR1, hGCM1 and hCYP19A1[11–17]. However, the role of miR-363-3p in lung cancer remain largely unknown.

PCNA is a critical eukaryotic replication accessory factor and plays crucial roles in DNA replication, DNA repair, the cell cycle, and apoptosis [18]. In the DNA damage repair and DNA replication system, In the DNA damage repair and DNA replication system, PCNA binds to the endonuclease, XPG (xeroderma pigmentosum complementation group G) and facilitates new DNA fragment resynthesis. PCNA itself is a cyclin protein that binds to p21 (CDK inhibitor) to promote cell cyclin and interacted with ING1 (inhibitor of growth 1) to regulate cell apoptosis. Therefore, PCNA can function as a bridging molecule that targets proteins with distinct roles in cell growth [19–21].

In a previous study, we found that miR-363-3p is down-regulated in lung cancer [22]. To identify a functional role for miR-363-3p in lung cancer, in this study we overexpressed miR-363-3p in lung adenocarcinoma cells. Our results demonstrate that miR-363-3p inhibits cell growth and tumor growth in nude mice. Furthermore, miR-363-3p can arrest S phase and enhance cell apoptosis in lung adenocarcinoma cells. Then, we identify PCNA as a direct target of miR-363-3p can rescind miR-363-3p-induced inhibition in lung adenocarcinoma cells. Our findings characterize a role for mR-363-3p in regulating the progression of lung adenocarcinoma and suggest a potential new therapeutic strategy.

## RESULTS

### miR-363-3p inhibits cell growth in lung adenocarcinoma cells and nude mice

**Figure 1:**
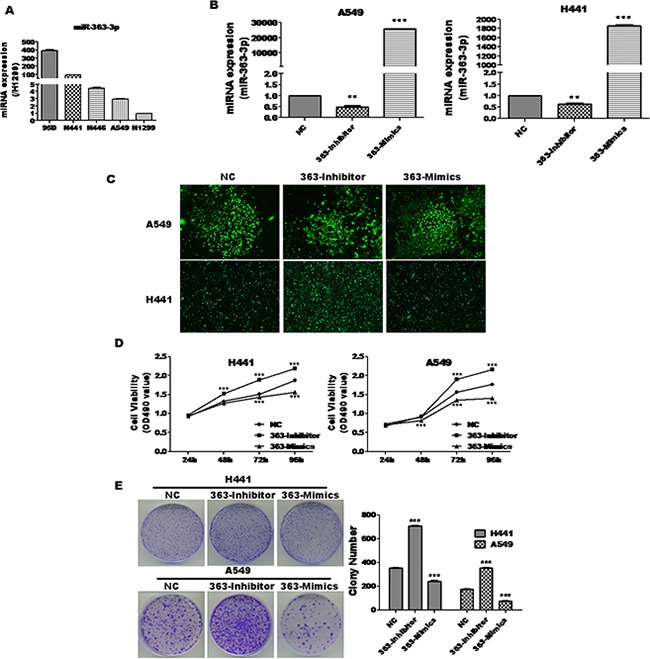
miR-363-3p significantly inhibited cell growth in A549 and H441 cells **A**. qRT-PCR analysis of miR-363-3p expression in five lung cancer cell lines. **B**. qRT-PCR analysis of miR-363-3p expression in various stable cell lines. U6 was used as an internal control. **C**. Fluorescence microscopy showing that A549 and H441 cells were successfully infected by lentivirus. **D**. A549 and H441 cells were infected with negative control (NC), miR-363-3p mimic (363-Mimics) or miR-363-3p inhibitor (363-Inhibitor) and seeded into 96-well culture plates at a density of 5 × 103 cells/well. Cell proliferation was subsequently assessed by MTT assay at the indicated times. **E**. 500 cells/well were seeded in plates and the medium was changed every 4 days. After 14 days, the plates were stained for the formation of cell colonies with crystal violet dye (left panel) and the number of colonies was counted (right panel). The data are expressed as the means ± SEM (n = 3), with results representative of 3 independent experiments shown. (** P < 0.01, *** P < 0.001 vs. NC).

In our previous study, we demonstrated that miR-363-3p is upregulated in lung cancer. To explore the potential role of miR-363-3p in various types of lung cancer, we firstly detected expression of miR-363-3p in various types of lung cancer cell lines (Figure 1A) and chose A549 (with relatively low miR-363-3p) and H441 cell (with relatively high miR-363-3p) as suitable cell lines for further study. To directly assess miR-363-3p function, we prepared stable A549 and H441 cell lines infected with a negative control non-targeting lentivirus (NC), a lentivirus expressing miR-363-3p inhibitor oligonucleotide (363-Inhibitor) or a lentivirus expressing exogenous miR-363-3p (363-Mimics). The efficiencies of the inhibitor and mimics in modulating miR-363-3p expression were confirmed by qRT-PCR (Figure 1B), and expression of the GFP marker gene confirmed high-level transduction efficiency (Figure 1C).

To investigate the effects of miR-363-3p on lung cancer cell proliferation, cell viability was measured at 24, 48, 72 and 96 hours, respectively after infection by MTT assay. As shown in Figure 1D, for both A549 and H441 cells, proliferation was strongly decreased by 363-mimics and strongly enhanced by 363-Inhibitor as compared to the NC. To further evaluate the inhibitory effect of miR-363-3p on the proliferation of non-small lung cancer cells, we performed clonogenic assays. Consistent with the results of the proliferation assay, the colony-forming efficiencies were significantly enhanced by 363-Inhibitor and reduced by 363-Mimic in both A549 and H441 cells (Figure 1E). These findings suggest that miR-363-3p suppresses the proliferation of lung cancer cells *in vitro*.

**Figure 2:**
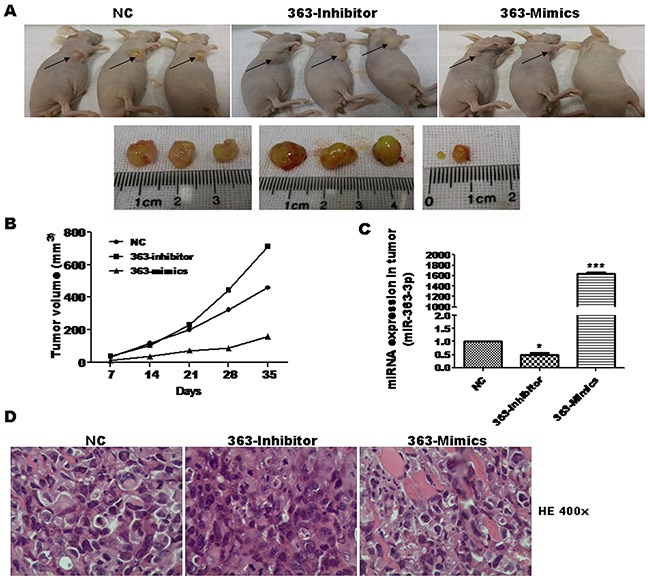
miR-363-3p significantly inhibited cell growth in nude mouse xenograft model **A**. Representative images of nude mice and tumor tumors isolated from a xenograft after 35 days. A549 cells were infected with the indicated lentiviruses prior to injection of 2×10^6^ cells in PBS into the nude mice (n = 6/group, two independent experiments). 35 days after cell injection, mice were sacrificed and the tumors were stripped from nude mice. **B**. Tumor volumes were measured with calipers every 7 days. Overexpression of miR-363-3p inhibited tumor growth, whereas inhibition of miR-363-3p stimulated tumor growth in a xenograft model. **C**. Expression of miR-363-3p in various tumor from the nude mouse xenograft model was examined by RT-QPCR. (*, p<0.05; ***, p<0.001 vs. NC). **D**. Representative H&E-stained sections of the tumor tissues was isolated from the nude mouse xenograft model. Magnification: 400 X.

To test the *in vivo* effect of miR-363-3p on tumor growth, we next used a tumor xenograft mouse model. Stably expressing A549 cells were subsequently injected into athymic nude mice, and differences in volume were observed for tumors harvested from mice sacrificed at day 35 (Figure 2A). The tumor volumes in mice injected with 363-Inhibitor cells were significantly larger than those of mice injected with the NC cells, while the tumor volumes in mice injected with 363-Mimics cells were significantly smaller (Figure 2B–2C). These results show that miR-363-3p can significantly inhibit the lung cancer cell growth *in vitro* and *in vivo*.

### miR-363-3p induces cell cycle arrest at S phase and promotes apoptosis

**Figure 3:**
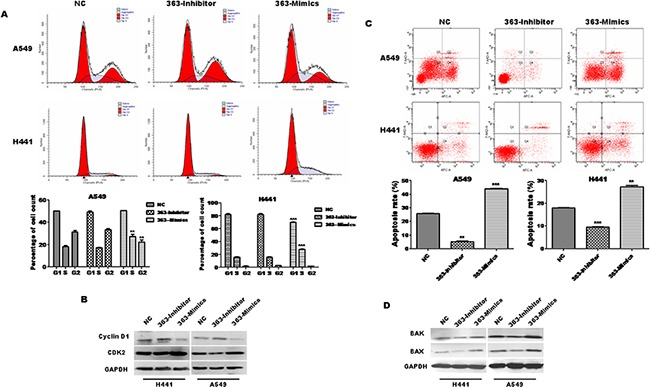
miR-363-3p induces cell cycle arrest and promotes cell apoptosis **A**. The representative FACS plots displayed differences in cell cycle phases of A549 and H441 cells infected with NC, 363-Inhibitor or 363-Mimics. Bar graphs show that miR-363-3p led to cell cycle arrest at S phase in A549 and H441 cells. The data is expressed as the mean ± SEM (n=3), with results representative of 3 independent experiments shown. **B**. Protein expression of Cyclin D1 and CDK2 in A549 cells was analysed by Western blotting. GAPDH was tested as a loading control. **C**. The cells were harvested and processed for apoptosis assay using the Annexin-APC/7-AAD Apoptosis Detection Kit. The cell distribution was analyzed by flow cytometry. Histograms from a representative experiment show the apoptotic effect of miR-363-3p on A549 and H441 cells. Overexpression of miR-363-3p increased nocodazol-induced apoptosis in A549 and H441 cells. In contrast, inhibition of miR-363-3p inhibited nocodazol-induced apoptosis in A549 and H441 cells. Bar graphs show that miR-363-3p promotes cell apoptosis and silencing of miR-363-3p reduces cell apoptosis in A549 and H441 cells. The data is expressed as the mean ±SEM (n=3), with results representative of 3 independent experiments shown. **D**. Protein expression of bax and bak in A549 cells was assessed by Western blotting. GAPDH was tested as a loading control. (**P<0.01, ***P<0.001,vs. NC).

Given that miR-363-3p suppresses the growth of lung adenocarcinoma cells, we asked whether this suppression may be caused by a block at a certain checkpoint in the cell cycle. Cell cycle distribution analysis for both A549 and H441 cell lines showed that 363-Mimics cells had statistically more S phase cells and statistically fewer G2 phase cells as compared to NC cells, though the cell cycle distribution was not statistically altered by miR-363-3p inhibitor (Figure 3A). Furthermore, 363-Mimics cells expressed less Cyclin D1 and more CDK2 than NC or 363-Inhibitor cells (Figure 3B). These results suggest that miR-363-3p inhibits the S/G2 transition, but not the G1/S transition, which could account for the block in cell cycle progression in 363-Mimic cells. On the basis of these findings, S/G2 cell cycle arrest in 363-Mimics lung adenocarcinoma cells may contribute to their poor proliferative ability.

To further examine whether miR-363-3p may induce apoptosis, we performed Annexin V-APC/7-AAD staining assays after induction of apoptosis with nocodazol in transduced cells. Our results demonstrate that inhibition of miR-363-3p significantly reduces cell apoptosis and overexpression of miR-363-3p has the reverse effect in both A549 and H441 cell lines (Figure 3C, ** P < 0.01). Furthermore, the expression of the apoptotic proteins Bak and Bax is reduced in 363-Inhibitor cells (Figure 3D). Collectively, these results suggest that miR-363-3p leads to a decrease in the proliferation of lung adenocarcinoma cells, which is associated with cell cycle arrest and apoptosis.

### miR-363-3p inhibits cell growth by regulating m-TOR and Erk signal pathway

**Figure 4:**
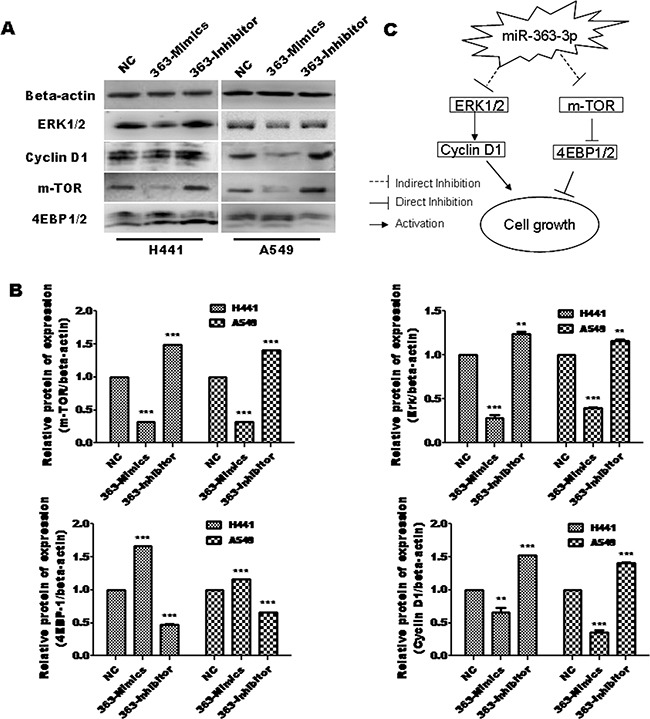
miR-363-3p regulates mTOR and Erk signal pathway **A**. Expression of miR-363-3p significantly affects protein expression of mTOR, 4EBP-1, Erk1/2 and Cyclin D1 as assessed by western blotting. **B**. Quantification of the results in panel A. The data are expressed as the means ± SEM (n = 3), with results representative of 3 independent experiments. (** p < 0.01 and *** p < 0.001 vs. NC). **C**. A mechanistic model showing suppressor cooperation between the mTOR and Erk1/2 pathway mediated by miR-363-3p.

To explore the mechanisms that are involved in miR-363-3p-dependent inhibition of cell proliferation, we examined upstream signaling molecules that are known to be involved in tumor cell growth. Western blotting assays showed that Erk1/2, which is a well-characterized upstream regulator of Cyclin D1, was downregulated in 363-Mimic A549 and H441 cell lines. In addition, m-TOR was significantly downregulated by miR-363-3p overexpression, while 4E-BP1, which known to be negatively regulated by m-TOR, had the opposite pattern of regulation by miR-363-3p (Figures 4A and 4B). Taken together, our findings suggest a model in which miR-363-3p regulates both the mTOR/Cyclin D1 pathway and the Erk-cyclin D1 pathway to inhibit tumor cell growth (Figure 4C).

### PCNA is a direct target gene of miR-363-3p

**Figure 5:**
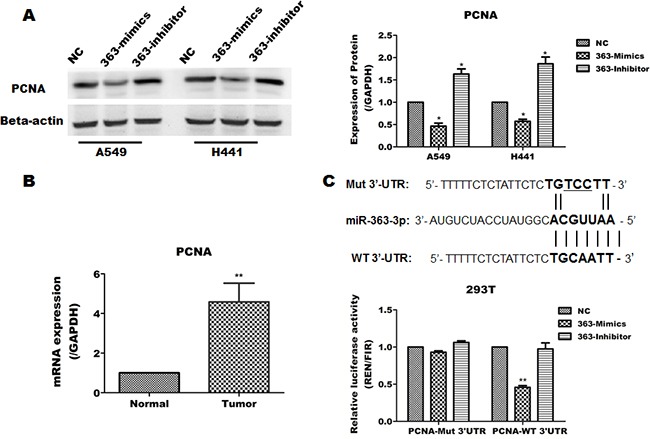
miR-363-3p inhibits the expression of PCNA by binding its 3′UTR **A**. The protein levels of PCNA was examined by Western blotting for three cell lines infected with either lenti-NC, lenti-363-mimic or lenti-363-inhibitor and quantification of the results in panel A. **B**. Expression of PCNA in lung cancer tissues compared with adjacent non-cancerous tissues. GAPDH was used as an internal control. (** P < 0.01 vs. Normal). **C**. The predicted miR-363-3p binding site in the 3′UTR of the PCNA mRNA was cloned into psiCHECK2 reporter vector, and a corresponding mutant was constructed. The constructs were then transfected into 293T cells, which were infected with either lenti-NC or lenti-363-mimics. The data are expressed as the means ± SEM (n = 3), with results representative of 3 independent experiments. (* p < 0.05 and ** p < 0.01 vs. NC).

To identify a functional target of miR-363-3p in NSCLC that may account in part for its anti-proliferative effects, we predicted miR-363-3p targets using the publically available databases Targetscan, MIRDB and DIANA-MICROT. Eight genes (TOB2, PTEN, RAB23, MYCBP2, PCNA, E2F3, TIMF1, MAP2K4) were selected as candidate target genes by the online program David Tools. We compared the expression of candidate target genes between lung cancer tissues and adjacent non-cancerous lung tissues from 50 patients and determined that the PCNA was expressed at higher levels in lung cancer tissues than in adjacent non-cancerous lung tissues (Figure 5B). Furthermore, overexpression of miR-363-3p significantly inhibited PCNA protein expression in A549 and H441 cells (Figure 5A).

To directly determine whether miR-363-3p can inhibit the expression of PCNA through its 3′UTR, we cloned the 3′UTR of PCNA into the plasmid psiCHECK-2 and prepared a specific mutant within the predicted miR-363-3p binding site as control. Luciferase assays showed that the expression of the wild-type, but not the mutant plasmid, was suppressed in 363-Mimics cells (Figure 5C). These results indicate that miR-363-3p negatively regulates PCNA by targeting a specific site in the PCNA 3′UTR. In addition, we identify that TOB2 was also an target of miR-363-3p (Supplementary Figure 1).

### PCNA can solely abrogate the effects of miR-363-3p

**Figure 6:**
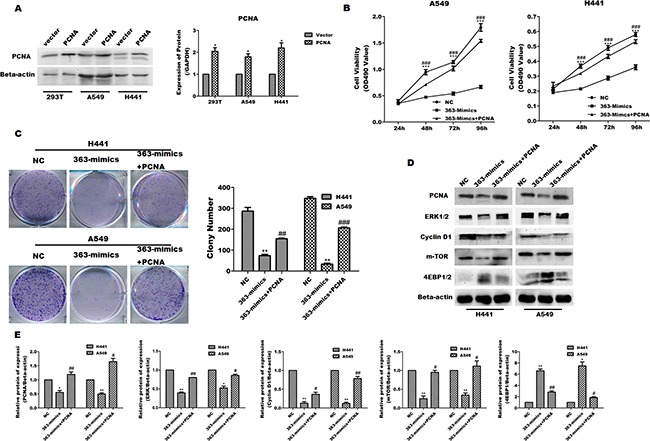
Overexpression of PCNA abrogates miR-363-3p induced inhibition in lung adenocarcinoma cells **A**. Expression of PCNA in A549 and H441 cells and quantification of the results in panel A. PCNA protein expression in cells transfected with a negative control (Vector), PCNA expression plasmid was analysed by western blot. **B**. Cell proliferation of A549 and H441 cells infected a negative control lentivirus (NC), and miR-363-3p-expressing lentivirus (363-mimics) or viruses expressing miR-363-3p and PCNA expression plasmid (363-mimics + PCNA) was assessed by an MTT assay at the indicated times. **C**. Colony formation ability was assessed. **D**. Expression of mTOR, 4EBP-1, Erk1/2 and Cyclin D1was assessed by western blotting in A549 and H441 cells infected a negative control lentivirus (NC), and miR-363-3p-expressing lentivirus (363-mimics) or viruses expressing miR-363-3p and PCNA expression plasmid (363-mimics + PCNA). **E**. Quantification of the results in panel D. Representative plates are shown, and the average colony numbers from 3 independent experiments are quantified. The data are expressed as the means ± SEM (n = 3), with results representative of 3 independent experiments. (** P < 0.01 vs. NC, ##P < 0.01 vs. 363-mimics).

To examine whether miR-363-3p may inhibit cell viability by targeting PCNA, we constructed overexpressed-vector to induced the expression of PCNA (Figure 6A). Similar to the results for miR-363-3p, the exogenous expression of PCNA in A549 and H441 cells can restore cell viability as assessed by MTT assay (Figure 6B) and retrieve cell growth as assessed by colony-formation assay after miR-363-3p-induced inhibition (Figure 6C). Moreover, overexpression of PCNA can relieve the inhibition of miR-363-3p on mTOR and ERK signaling pathways (Figure 6D). These results are consistent with the possibility that miR-363-3p inhibits tumor cell growth by targeting PCNA. PCNA overexpression in A549 and H441 cells significantly promote cell viability and abrogate miR-363-3p-induced inhibition. Taken together, these results suggest that PCNA is a direct target of miR-363-3p and contribute to the miR-363-3p-dependent inhibition of A549 and H441 cell growth. In addition, TOB2, the other target of miR-363-3p, can't abrogate the effects of miR-363-3p in A549 and H441 cells, and we also detected relationship between Tob2, mTOR and ERK signaling pathways. (Supplementary Figure 2, Supplementary Figure 3, Supplementary Figure 4 and Supplementary Table 1).

## DISCUSSION

Our knowledge of the molecular pathogenesis of lung cancer has increased tremendously over the past decade, The deregulation of miRNAs has been described in many types of human cancers have been identified [25, 26], Numerous studies indicate that miRNAs may have dual functions in tumor progression as either a oncogenes or suppressors [27, 28]. In our previous study, we identified miR-363-3p was a potential tumor suppressor in lung cancer [22]. A previous study of liver cancer indicated that miR-363-3p can reduced tumorigenesis by inhibiting the G1 to S phase transition [11]. Additionally, miR-363-3p was a strong predictor of favorable prognosis and was expression of miR-363-3p significantly associated with conventional prognostic factors in lung cancer [14]. These report suggest that miR-363-3p may be a key molecule in lung carcinogenesis. However, prior to this study, the function of miR-363-3p in lung cancer remained unknown. In this study, we demonstrated that miR-363-3p could inhibit cell proliferation and colony formation, induced cycle arrest and promoted cell apoptosis of lung adenocarcinoma cells. Moreover, tumorigenicity assays in nude mice demonstrated that miR-363-3p suppresses tumorigenesis of lung adenocarcinoma cells *in vivo*. Take together, we provide evidence that miR-363-3p function as a tumor suppressor in lung adenocarcinoma.

The cell cycle is driven by the alternative expression or degradation of different cyclins which exert their function by binding to different CDKs [29, 30], Cyclin D is activated by binding to CDK4/6 during the cell cycle progression, while the Cyclin E/CDK2 complex pushes cell cycle through the G1/S transition and the Cyclin A/CDK2 complex drives cells through the S phase [31, 32]. Alterations in the machinery that controls the G1/S transition are frequently observed in many types of human cancers [33, 34]. Our data showed that enforced expression of miR-363-3p reduced protein expression of cyclin D1 and increases the protein expression of cdk2, which explains why cells accumulated in the S phase, rather than the G1/S transition,when miR-363-3p is overexpressioned These data indicated that miR-363-3p disturbs the mitotic machinery by interfereing at several checkpoint molecules, which result in a growth blockade on lung adenocarcinoma cells.

Apoptosis is another crucial mechanism underlying the regulation of tumor growth. In the current study, repeated experiments indicated that miR-363-3p increases nocodazol-induced apoptosis in lung adenocarcinoma cells. Moreover, we found miR-363-3p increased the accumulation of two well established pro-apoptotic marker, Bax and Bak, which is consistent with previous findings [35, 36]. Take together, these data demonstrated that miR-363-3p inhibited tumor growth by increasing cell apoptosis and inducing cell cycle arrest.

Preclinical studies demonstrated that the RAS/RAF/ERK and PI3K/AKT/mTOR pathway are potential therapeutic targets for various types of cancer [37, 38]. The combination of mTOR inhibitor and ERK inhibitor promotes apoptosis and tumor regression in mouse models of colorectal cancers [39]. Our results showed that enforced expression of miR-363-3p inhibits the accumulation of mTOR and ERK and regulates expression of 4E-BP1 and cyclin D1, which are downstream molecules of mTOR and ERK pathway. These data indicated miR-363-3p suppressed tumor growth through inhibiting mTOR /ERK pathway. Because no miR-363-3p binding sites were predicted within the mTOR and ERK genes, it is likely that the suppression of these pathways occurs indirectly through other miR-363-3p target molecules.

microRNAs regulate cell proliferation by modulating the expression of target genes [24]. In this study, as a predic target gene of miR-363-3p, althouth we found expression of TOB2 was also significantly decreased after infecting with 363-Mimics, and TOB2 knockdown inhibited cell viability, but TOB2 overexpression in A549 and H441 cells did not significantly promote cell viability and could not abrogate miR-363-3p-induced inhibition, see Supplementary Figure 2 and Supplementary Figure 3C, indicating the role of miR-363-3p for tumor growth could not be explained by TOB2, since a miRNA could have multiple target gene and complex signal network. Furthermore, Our data demonstrate that PCNA is a target gene of miR-363-3p. Previous studies suggested that PCNA regulates cell cycle and apoptosis and interacts with its partner protein to promote cell proliferation *in vitro* and *in vivo* [18, 20, 21]. In this study, we found that PCNA is a direct target genes to miR-363-3p in lung adenocarcinoma cancer, and exogenous PCNA expression significantly affect the proliferation of lung adenocarcinoma cells and abrogate miR-363-3p-induced inhibiton. These data indicated miR-363-3p regulates cell proliferation by targeting PCNA in lung adenocarcinoma cancer.

In conclusion, miR-363-3p is down-regulate in lung cancer tissues and inhibits tumor growth by inducing cell cycle arrest and promoting apoptosis in lung adenocarcinoma. Our study identifies miR-363-3p as a potential target of lung adenocarcinoma therapy, which may help to establish a novel strategy for lung adenocarcinoma therapy.

## MATERIALS AND METHODS

### Cell lines and tissue samples

The human lung carcinoma cell lines A549 and H441 were purchased from the Shanghai Cell Institute Country Cell Bank (Shanghai, China). These cell lines were cultured in DMEM or RIPM1640 supplemented with 10% heat-inactivated FBS (GIBCO, USA), 100 U/ml penicillin G and 100 μg/ml streptomycin (Sigma-Aldrich, MO) at 37°C in a humidified 5% CO_2_ atmosphere.

Tissue samples were obtained from the Department of Cardiothoracic Surgery, Affiliated Hospital of Guangdong Medical College. After surgery removal, all tissue samples were immediately frozen in liquid nitrogen and stored at−70°C until use. We analyzed all samples histologically to assess the amount of tumor component (at least 80% tumor cells) and the quality of material. Normal tissues were defined histologically confirmed by using the classical pathology approaches (the distance from the primary tumor was >5 cm), and observation by a pathologist. We retrospectively reviewed the medical records of patients, and available clinical and follow-up information in the Affiliated Hospital of Guangdong Medical Collage (Zhanjiang, China). This study was approved by the Affiliated Hospital of Guangdong Medical College Ethics Committee (No:PJ2012132), and carried out under approved guidelines. Patients were told that tumor tissue from them were used for medical research and confirmed “informed consent” for this project.

### Reagents and antibodies

Oligonucleotides, including negative control miRNA, miR-363-3p mimics, inhibitor oligonucleotides and corresponding lentivirus productions were synthesized by GenePharma (Shanghai, China). Oligonucleotide transfection were performed using Lipofectamine 2000 reagent (Invitrogen, USA) and lentivirus infection according to the manufacturer's protocol. The antibodies used in this study were β-actin (Santa Cruz, USA), β-tublin (Earth, USA), GAPDH, Cyclin D1, mTOR, ERK1/2, 4E-BP1, CDK2, BAX, BAK (Cell Signaling, USA), PCNA (Sangon Biotech, China) (Supporting Information Table 2).

### RNA extraction and quantitative real-time polymerase chain reaction (qRT-PCR)

miRNA extraction is as same as our previous study [23], the detail processe is as following: Firstly, total RNAs were extracted using the TRIzol reagent according to the manufacturer's protocol (Invitrogen, USA). The miRcute miRNA First-Strand cDNA Synthesis Kit and miRcute miRNA qPCR detection kit were purchased from TIANGEN BIOTECH (Beijing, China) for assays to quantify mature miR-363-3p with U6 small nuclear RNA as an internal control. The primers were designed and Synthesized by TIANGEN BIOTECH (Beijing, China). Data analysis was performed using the 2^−△△Ct^ method [24].

### Plasmid construction

The 3′UTR and CDS sequence of PCNA were amplified from A549 cell total RNA by RT-PCR. The 3′UTR sequence was subcloned into psiCHECK-2 vector (Promega) and the CDS sequence was subcloned into pCDNA3.1+ vector (Promega). All constructs were verified by sequencing.

### Creation of miR-363-3p stable cell lines

A549 or H441 cells were infected with recombinant lentivirus in the presence of 5 μg/ml polybrene (Sigma, USA). Infected cells were selected for 14 days in the presence of 2 μg/ml puromycin (Sigma, USA). Expression of miR-363-3p in infected cells was verified by quantitative reverse transcription PCR (qRT-PCR).

### MTT assays

Briefly,5000 cells/well were plated in triplicate in 96-well plates, and MTT assays were performed according to the manufacturer's protocol. The absorbance at 490 nm was measured using a multiwell plate reader.

### Colony formation assays

For colony formation assays, A549 or H441 cells (500 cells/well) were seeded in a 60 mm plate in triplicate. After 14 days incubation, plates were gently washed with PBS and stained with 0.1% crystal violet. Colonies with more than 50 cells were manually counted. Plating efficiency was calculated by dividing the number of colonies formed in the treated group by that in the control group.

### Tumorigenicity assays in nude mice

This study was carried out in strict accordance with the recommendations in the Guide for the Care and Use of Laboratory Animals of Guangdong Medical College. The protocol was approved by the Committee on the Ethics of Animal Experiments of Guangdong Medical College (Permit Number: SCXK(Guangdong)2013-0008). All surgery was performed under sodium pentobarbital anesthesia, and all efforts were made to minimize suffering. Six-week-old female athymic nude mice weighing 20~22 g were subcutaneously injected in the right armpit region with 1.5×10^6^ cells in 0.1 ml of PBS. Three groups of mice (n = 6/group) were tested. Group 1 (NC) was injected with A549 cells infected with negative control miRNA; and group 2 (363-inhibitor) was injected with A549 cells infected with miR-363-3p inhibitor; group 3 (363-mimics) was injected with A549 cells infected with miR-363-3p mimics. Tumor sizes were measured every 7 days with calipers. Tumor volume were calculated with the formula: (L × W2)/2, where L is the length and W is the width of the tumor.

### Cell cycle analysis

For cell cycle analysis, 2 × 10^5^ cells were plated in a 60 mm culture plate and grown for 24 h. The cells were then incubated with 2.5 mg/ml hydroxyurea (Sigma-Aldrich) for 24 h to synchronize cells at the G1/S boundary, followed by infection with control or miR-363-3p expressing lentiviruses; 48 h later, cells were subsequently analyzed by flow cytometry (BD Biosciences, USA) and cell cycle analysis used cell cycle staining (Liankebio, China) and were performed according to the manufacturer's protocol.

### Cell apoptosis analysis

For cell apoptosis analysis, A549 and H441 cells were seeded in 6-well plates (1.5×10^5^ cells/well) and grown for 24h, then incubated with 10 ng/ml nocodazol (Sigma-Aldrich) for 16h to induce cells to tend apoptosis, and harvested and washed with cold PBS. The cell surface phosphatidylserine in apoptotic cells was quantitatively estimated by using an Annexin V-APC/7-AAD double staining apoptosis detection kit (Liankebio, China) according to manufacturer's instructions. The percentage of apoptotic cells was analyzed by flow cytometry. Triplicate experiments with triplicate samples were performed.

### Western blotting

Cells were harvested and resuspended in SDS buffer (Beyotime, Shanghai, China) for preparation of total protein extracts. Western blotting analysis was performed according to the antibody (Cell Signaling, USA) manufacturer's protocol.

### miRNA target prediction

The online program Targetscan, MIRDB and DIANA-MICROT were used to predict potential targets of miR-363-3p. We analyzed potential targets and selected eight genes (TOB2, PTEN, RAB23, MYCBP2, PCNA, E2F3, TIMF1, MAP2K4) as candidate target genes using the online program David Tools, and expression of TOB2 and PCNA was significantly decreased after infecting with 363-Mimics.

### Luciferase assays

293T cells were seeded in 96-well plates at 6,000 cells per well the day before transfection. A mixture of 100 ng psi-PCNA 3′UTR vector and 200 ng of NC or miR-363-3p mimics was transfected into 293T cells with Lipofectamine 2000. Forty-eight hours later, Firefly and Renilla luciferase activities were measured with a Dual-Luciferase Reporter System (Promega, USA) according to the manufacturer's protocol.

### Statistical analysis

Results are presented as means ± standard error of the mean (SEM). Differences between means were analyzed using a two-tailed Student's t-test and considered statistically significant when p < 0.05. All statistical analysis was performed using SPSS 17.0 software (SPSS Inc., USA).

## SUPPLEMENTARY MATERIALS FIGURES AND TABLES


