# Supplementary material for: miR-363-3p inhibits tumor growth by targeting PCNA in lung adenocarcinoma
Source: Oncotarget. 2017 Feb 17;8(12):20133–44. doi: 10.18632/oncotarget.15448 (PMC5386750; doi:10.18632/oncotarget.15448)
Supplement: Supplementary file 1 [file oncotarget-08-20133-s001.pdf]

miR-363-3p inhibits tumor growth by targeting PCNA in lung adenocarcinoma

SUPPLEMENTARY FIGURES AND TABLES

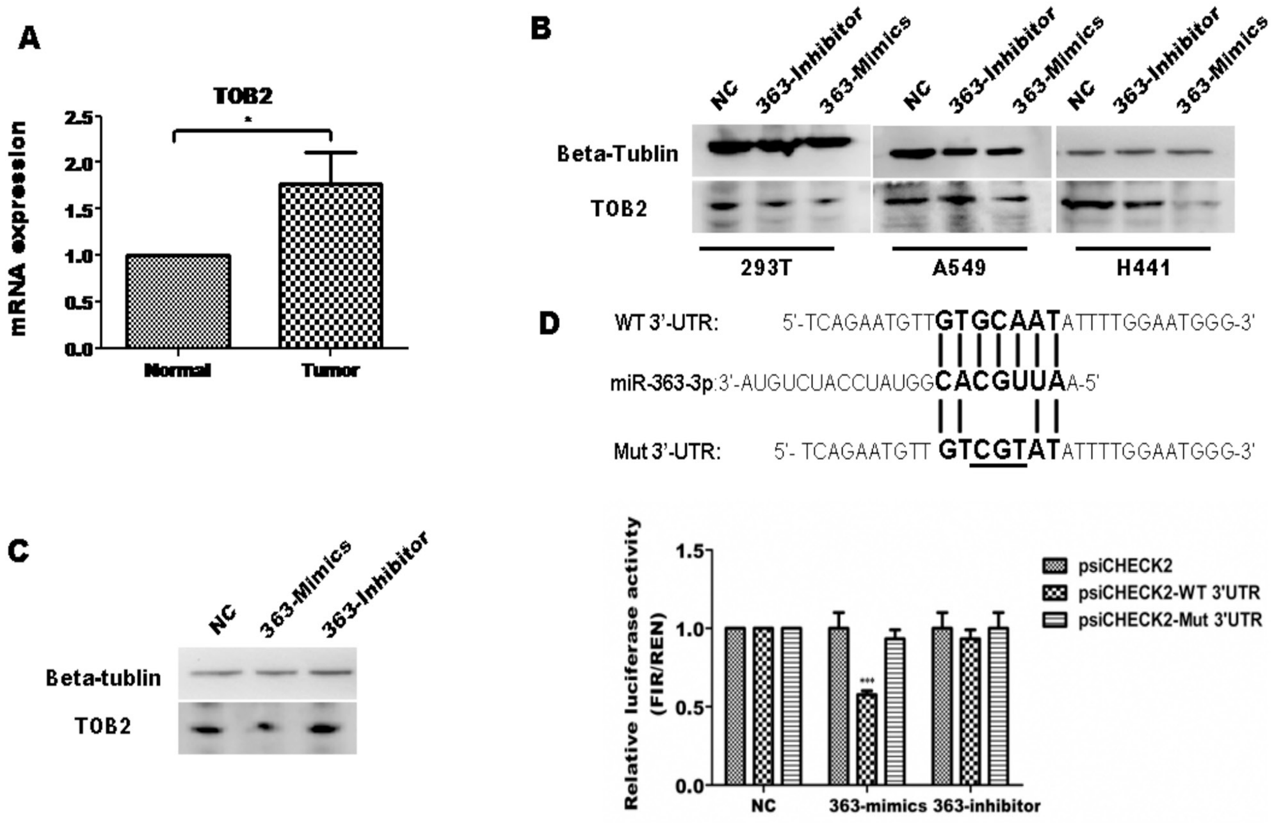

Supplementary Figure 1: A. Expression of Tob2 in lung cancer tissues compared with adjacent non-cancerous tissues. GAPDH was used as an internal control. (* $P < 0.005$ vs. Normal). **B.** The protein levels of Tob2 were examined by Western blotting for three cell lines infected with either lenti-NC, lenti-363-mimic or lenti-363-inhibitor. **C.** Expression of Tob2 in tumors from the nude mouse xenograft model was examined by Western blotting. **D.** The predicted miR-363-3p binding site in the 3'UTR of the Tob2 mRNA was cloned into psiCHECK2 reporter vector, and a corresponding mutant was constructed. The constructs were then transfected into 293T, A549 and H441 cells, which were infected with either lenti-NC, lenti-363-mimic or lenti-363-inhibitor. The data are expressed as the means ± SEM (n = 3), with results representative of 3 independent experiments. (** $p < 0.01$ and *** $p < 0.001$ vs. NC).

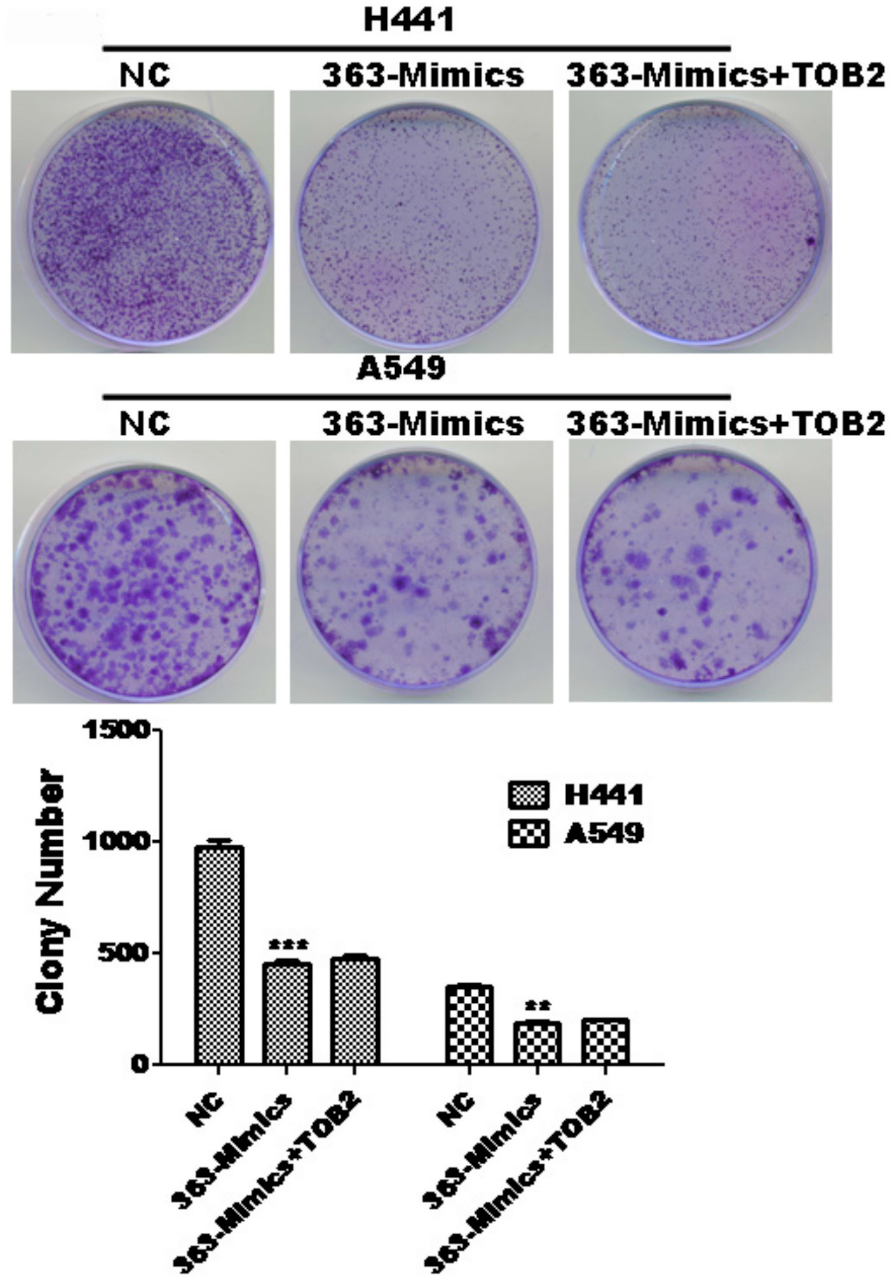

Supplementary Figure 2: Overexpression of Tob2 could not completely abrogate miR-363-3p induced inhibition of A549 and H441 cell growth. Overexpression of miR-363-3p suppressed Cell proliferation by colony formation assays, and both Overexpression of miR-363-3p and Tob2 could not restored ability of cell proliferation. The data is expressed as the mean ± SEM (n=3), with results representative of 3 independent experiments shown. (**, P<0.01, vs. NC).

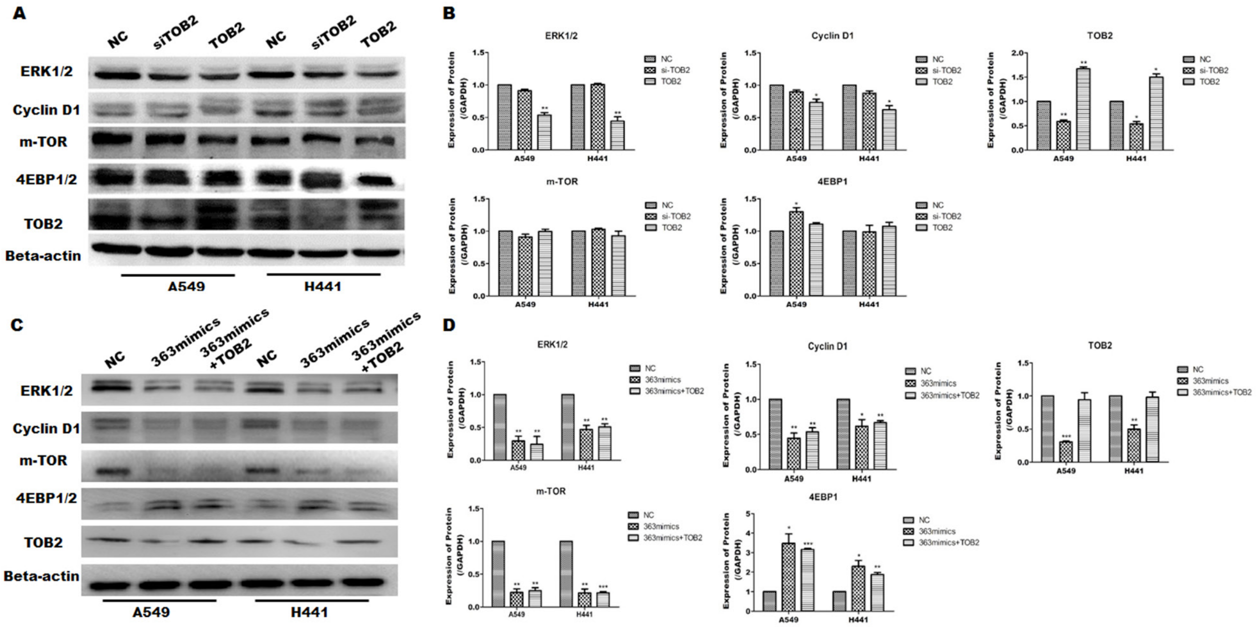

Supplementary Figure 3: A. Silencing Tob2 or overexpressed tob2 in A549 and H441 cell, and expression of mTOR-4EBP1 pathway and the Erk-cyclin D1 pathway was examined by Western blotting. **B.** Quantification of the results in panel A. **C.** Both Overexpression of miR-363-3p and Tob2 in A549 and H441 cell, and tob2 could not restored expression of mTOR-4EBP1 pathway and the Erk-cyclin D1 pathway. **D.** Quantification of the results in panel C. The data is expressed as the mean ± SEM (n=3), with results representative of 3 independent experiments shown. (*,P<0.01;**, P<0.01;***, P<0.01 vs. NC).

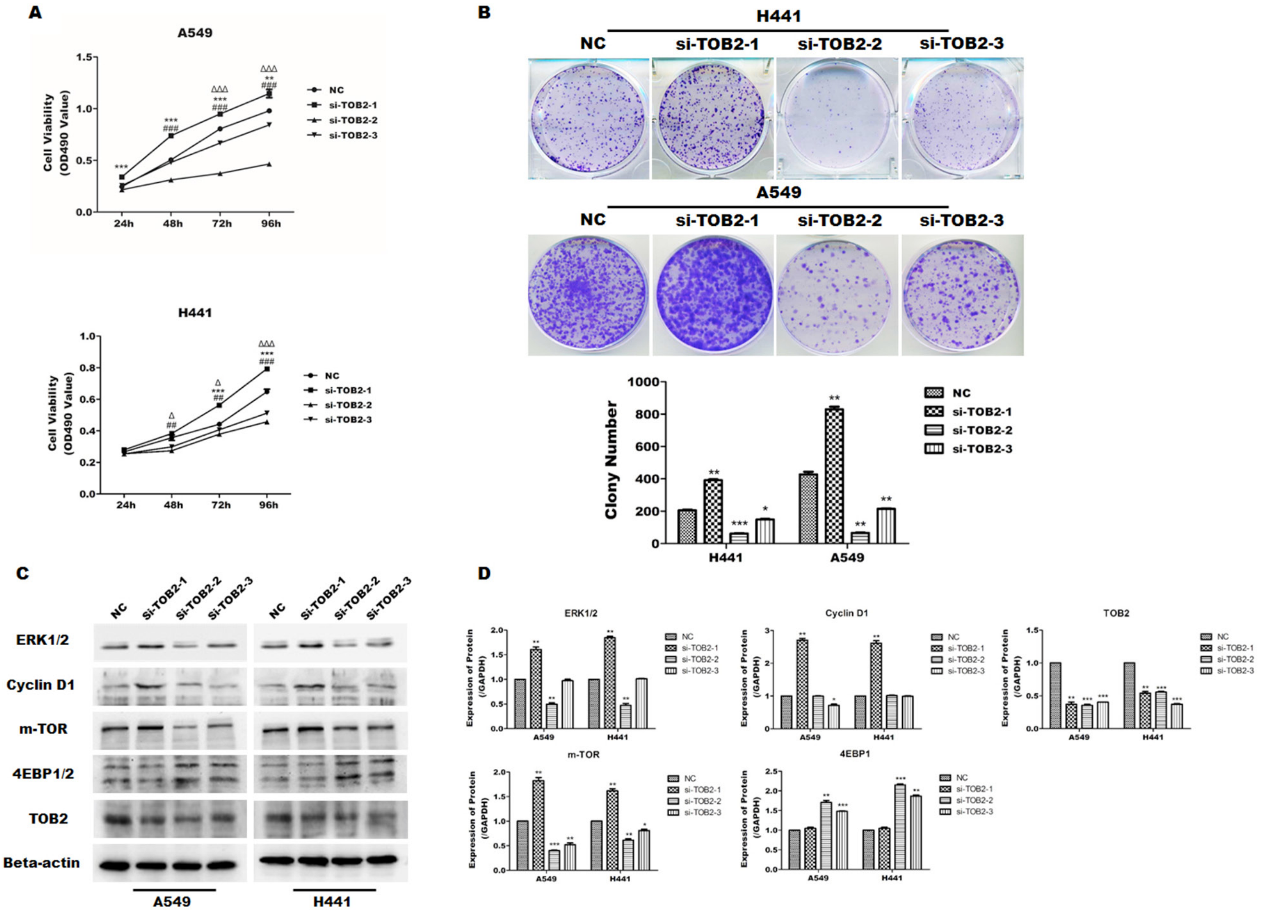

Supplementary Figure 4: A549 and H441 cells were infected with negative control (NC), siTOB2-1 (Silencing tob2), siTOB2-2 (Silencing tob2) or siTOB2-3 (Silencing tob2) and A. seeded into 96-well culture plates at a density of 5 × 103 cells/well. Cell proliferation was subsequently assessed by MTT assay at the indicated times. (** $P < 0.01$, *** $P < 0.001$, siTOB2-1 vs. NC; ##$P < 0.01$, ### $P < 0.001$, siTOB2-2 vs. NC; ΔΔ $P < 0.01$, ΔΔΔ $P < 0.001$, siTOB2-3 vs. NC). **B.** 500 cells/well were seeded in plates and the medium was changed every 4 days. After 14 days, the plates were stained for the formation of cell colonies with crystal violet dye (upper panel) and the number of colonies was counted (lower panel). The data are expressed as the means ± SEM (n = 3), with results representative of 3 independent experiments shown. (** $P < 0.01$, *** $P < 0.001$ vs. NC).

Supplementary Table 1: Sequences of siRNA for Tob2

Name	Sequences(5'-3')
siTOB2-1	GCCCUGAACUUCAUCAUCUTT
	AGAUGAUGAAGUUCAGGGCTT
siTOB2-2	CCAAAUUUGGCUCCACUAATT
	UUAGUGGAGCCAAAUUUGGTT
siTOB2-3	CCUGGAGAAGACACCCUUUTT
	AAAGGGUGUCUUCUCCAGGTT
siTOB2	Purchased from Santa Cruz

Supplementary Table 2: Information of antibody

Name	host species	company	catalogue	dilution	monoclonal(M)/polyclonal(P)
β-actin	Rabbit	Santa Cruz	SC-130656	1:500	M
β-tublin	Mouse	Earth	E021040	1:500	M
GAPDH	Rabbit	Cell Signaling	2118	1:1000	P
Cyclin D1	Rabbit	Cell Signaling	2978	1:500	P
mTOR	Rabbit	Cell Signaling	2983	1:500	P
ERK1/2	Rabbit	Cell Signaling	4695	1:500	P
4E-BP1	Rabbit	Cell Signaling	9644	1:500	P
CDK2	Rabbit	Cell Signaling	2546	1:500	M
BAX	Rabbit	Cell Signaling	5023	1:500	P
BAK	Rabbit	Cell Signaling	6947	1:500	P
PCNA	Rabbit	SangonBiotech	D220014	1:1000	P
TOB2	Mouse	Abcam	ab56760	1:500	M
